# One year experience of swine dermal non-crosslinked collagen prostheses for abdominal wall repairs in elective and emergency surgery

**DOI:** 10.1186/s13017-015-0023-8

**Published:** 2015-07-01

**Authors:** Giulia Montori, Federico Coccolini, Roberto Manfredi, Marco Ceresoli, Luca Campanati, Stefano Magnone, Michele Pisano, Elia Poiasina, Gabriela Nita, Fausto Catena, Luca Ansaloni

**Affiliations:** Unit of General and Emergency Surgery, Papa Giovanni XXIII Hospital, P.zza OMS 1, 24128 Bergamo, Italy; General surgery department, Ospedale Maggiore, Parma, Italy

**Keywords:** Biological mesh, Ventral hernia, Abdominal wall repair, Swine dermal collagen, Open abdomen, Laparostomy, Emergency surgery

## Abstract

**Introduction:**

The approach to the abdominal wall surgical repair is dramatically changed in the last years. This study evaluates our institutional outcomes about the usage of biological meshes for abdominal wall repair in different setting: in elective surgery, in emergency surgery and in abdominal wall repair following open abdomen (OA) procedure.

**Methods:**

A database was prospectively conducted (January–December 2014) and data were reviewed for patients who underwent to an abdominal wall reconstruction with swine dermal non-cross linked collagens prostheses either in elective or emergency setting, and following OA/laparostomy procedure. Demographic data, co-morbidities, indications for surgery, intra-operative details, post-operative complications and outcome (peri-operative, 3, 6, 9-months) were analyzed.

**Results:**

A total of 30 cases were reported: 9 in elective surgery (Group 1), 4 in emergency surgery (Group 2) and 17 with abdominal wall closure following OA management (Group 3). Two meshes were removed: 1 in the Group 1 and 1 in the Group 3. During follow-up only one patient in the Group 3 had a recurrence of the incisional hernia. Mortality rate was 11.1 % at 3 months in Group 1, 0 % in the Group 2, and 29.4 % in peri-operative period in the Group 3.

**Conclusions:**

The use of non-cross linked biological meshes can be safe and versatile in different situations from elective to emergency surgery, and also for the reconstruction of the abdominal wall after OA procedure, with an acceptable recurrence and mortality rate.

## Background

Abdominal wall hernia is one of the most common conditions encountered by general surgeons. Procedures to repair abdominal wall defects are more than 990.000 each year in USA, with an estimated 567.000 of them performed in emergency [1, 2]. Similar data are reported in other Western countries [3]. In most cases emergency hernia repair are performed in adults over 65 years old [2]. These age-related increasing rates of emergency hernia repair are associated with increased morbidity and mortality risks [2]. In these particular situations the use of biological materials can be safer than synthetic meshes [4]. Actually in the late 1990s, biologic materials for hernia repair were introduced as a possible biocompatible material to support “in vivo” tissue regeneration to restore the physiological function of the abdominal wall [4, 5]. Although these biomaterials enacted a new conceptual approach in abdominal wall reconstruction [5, 6], the main reported problems related with the use of biological meshes are: wound infections, seromas, fistula formation, recurrence of the defect (25–28 % in grade 3–4 of the Ventral Hernia Working Group (VHWG) classification [4]) and costs [7, 8]. Nevertheless definitive conclusions about the use of these biological materials are difficult to drawn due to: lack of adequate follow-up, use either of different surgical techniques and/or different types of biological meshes and absence of high levels of evidence studies [7, 8]. However there are many advantages in using biological meshes compared to the synthetic prostheses: less prosthetic erosions and/or fistulization, less chronic pain, and minor risk of infection [9].

Porcine dermal prostheses are manufactured by collagenic tissue harvesting, followed by a variety of patented chemical treatments and delipidation processes [10]. Although natural cross-links exist in native collagen and function to stabilize the structure of the collagenic proteins, giving mechanical strength and protection from collagenase [10], however some prostheses undergo to an additional phase chemical cross-linking process to increase the collagen strength [10]. The addition of chemical cross-linking between the collagen chains seems to further reduce the bacterial and host collagenase enzymes activity, slowing the degradation process of the prostheses. For this reason the indications report in literature suggest a use of cross-linked mesh in more contaminated field [11]. However some authors reports that porcine biologic non-cross-linked mesh has been used to support the repair of abdomen wall with a hernia recurrence rates of 7 % in case of underlay position, while in the cross-linked porcine mesh group infection and explantation rates were relatively higher [9]. These data indicate that there is currently no ideal biologic mesh for complex hernia repair and the candidate for biologic mesh implantation has not been clearly defined yet. A Randomized Control Trial (SIMBIOSE study: use of biological mesh versus standard wound care in infected incisional ventral hernias) is ongoing to evaluate the efficacy of biologic mesh in infected field [12].

This study evaluates our institutional outcomes about the usage of biological meshes for abdominal wall repair in three different setting: in elective surgery, in emergency surgery and following open abdomen (OA)/laparostomy procedure.

## Methods

An analysis was performed on a prospectively maintained database from January 1^st^, 2014 to December 31^st^, 2014 of all patients who underwent primary or recurrent hernia repair with the usage of swine dermal collagens prostheses at Papa Giovanni XXIII Hospital (Bergamo, Italy). Enclosed patients were divided into 3 groups: abdominal wall reconstruction in elective surgery (Group 1), in emergency surgery (Group 2) and following OA/laparostomy management (Group 3). In all patients were used 1.4 mm thick, non-cross linked swine dermal collagen prostheses, with different dimensions (8*14, 10*20, 25*18, 20*30) (Protexa™, Tecnoss, Giaveno, Italy), fixed to the fascia with resorbable PDS 1 or 0. In case of elective surgery and in clean field the antibiotic prophylaxis used was Cefazolin 2 g, in the dirty and contaminated field the antibiotic theraphy was accord to the WSES guideline for the management of the intra-abdominal infection (IAI) [13]. Patients data were collected and analyzed evaluating demographic characteristics, co-morbidities, surgical indications, intra-operative details, complications (according to the Dindo-Clavien classification [14]) and outcome (peri-operative, at 3, 6, 9 months). Incisional hernia was classified according to the VHWG classification [4]. In case of septic implant, the peritonitis severity was evaluated according to the Mannheim Peritonitis Index (MPI) at the first laparotomy and at every subsequent abdominal exploration if indicated [15]. The severity of the trauma was calculated according to the Injury Severity Score (ISS) [16]. Balthazar score was used to evaluate the severity of pancreatitis [17]. The OA was classified according to the Björck classification [18].

## Results

Thirty patients undergoing abdominal wall reconstruction with indication for biologic mesh placement and with swine dermal non-crosslinked collagen prostheses implant were enrolled in the study period. Of these patients, 9 were in Group 1 (Table 1), 4 in Group 2 (Table 2), and 17 in Group 3 (Table 3). According to the Hernia Grading System [4] 5 of the 30 patients presents a clean surgical site but high comorbidities, 21 patients have a contaminated field, and four patients have a dirty surgical site.

**Table 1:** Patients characteristics underwent to abdominal wall repair in elective surgery

Characteristics (*N* = 9)	Value	*N* (%)
Gender	Male	5 (55.6)
	Female	4 (44.4)
Age	Mean (SD), yrs	68.3 (±9.3)
	Median (range), yrs	68 (53–83)
Surgical indications	Incisional hernia	9 (100)
Comorbidities^a^	Immunodepression	4
	Abdominal aortic aneurism	1
	Diabetes	1
	Malignancy	3
	Cardiopathy	2
	BPCO	2
	None	3
Indications for the positioning of the prostheses	Colic anastomosis	6 (66.7)
	Previous prosthetic infection	1 (11.1)
	Intraabdominal infection	2 (22.2)
Prosthetic position	Onlay	0 (0)
	Inlay	0 (0)
	Retromuscolar	6 (66.7)
	Preperitoneal	0 (0)
	Intraperitoneal	3 (33.3)
Hospital stay	Mean (SD), dys	21 (±21)
	Median (range), dys	14 (7–70)
Mortality rates	At 1 month	0
	At 3 months	1 (11.1)
	At 6 months	0
	At 9 months	0

(*SD* standard deviation; *pts* patients, *yrs* years, *BPCO* bronchopneumopathy chronic obstructive)

^a^More than one patient has more than one comorbidity

**Table 2:** Patients characteristics underwent to abdominal wall repair in emergency surgery

Characteristics (*N* = 4)	Value	*N* (%)
Gender	Male	2 (50)
	Female	2 (50)
Age	Mean (SD), yrs	76.5 (±9.4)
	Median (range), yrs	75.5 (66–89)
Surgical indications	Complicated incisional hernia	1 (25)
	Diverticular perforation and incisional hernia	1 (25)
	Eventration and ileo anastomosis	1 (25)
	Recto-vaginal fistula and incisional hernia	1 (25)
Comorbidities^a^	Corticosteroid therapy	2
	Cardiovascular disease	2
	Inflammatory bowel disease	1
	Cerebrovascular disease	1
	None	2
Prothetic position	Onlay	1 (25)
	Inlay	1 (25)
	Retromuscolar	2 (50)
	Preperitoneal	0 (0)
	Intraperitoneal	0 (0)
Hospital stay	Mean (SD), dys	21.5 (±16.8)
	Median (range), dys	16.5 (7–45)
Mortality rates	At 1 month	0 (0)
	At 3 months	0 (0)
	At 6 months	0 (0)
	At 9 months	0 (0)

(*SD* standard deviation, *pts* patients, *dys* days, *yrs* years)

^a^More than one patient has more than one comorbidity

**Table 3:** Patients characteristics underwent to abdominal wall repair in open abdomen

Characteristics (*N* = 17)	Value	*N* (%)
Gender	Male	8 (47)
	Female	9 (53)
Age	Mean (SD), yrs	62.6 (±18.6)
	Median (range), yrs	72 (18–79)
Surgical indications to open abdomen	Intra-abdominal sepsis and/or post-operative complications	7 (41.2)
	Pancreatitis	1 (5.8)
	Trauma	3 (17.6)
	Vascular complications	2 (11.8)
	Intestinal ischemia	4 (23.6)
Comorbidities^a^	Corticosteroid therapy	2
	Cardiopathy and vasculopathy	9
	Malignancy	6
	Infectious diseases, immunodepression	5
	Abdominal aortic aneurism	4
	Hemodialisis	1
	Diabetes	1
	None	3
Provenience of the pts	ICU	8 (47)
	Ward	5 (29.5)
	Emergency department	4 (23.5)
ISS (*N* = 3)	ISS mean (SD)	38.3 (±14.3)
	ISS median (range)	35 (26–54)
Bathazar score (*N* = 1)	E	
MPI (*N* = 14)	MPI mean (SD)	26.6 (±8.8)
	MPI median (range)	26 (15–43)
	<21	4 (28.6)
	21–29	5 (35.7)
	>29	5 (35.7)
Bjorck classification at the first laparotomy	Ia	7 (41.4)
	Ib	4 (23.5)
	IIa	0 (0)
	IIb	6 (35.1)
	III	0 (0)
	IV	0 (0)
Bjorck classification at the closure	Ia	13 (76.6)
	Ib	0 (0)
	IIa	3 (17.6)
	IIb	0 (0)
	III	0 (0)
	IV	1 (5.8)
Causes of OA	Prevention of ACS	12 (70.6)
	Treatment of ACS	5 (29.4)
Type of OA	NPWT	16 (94.2)
	Skin closure	1 (5.8)
Closure abdominal wall	Mean (SD), dys	6.1 (±3.5)
	Median (range), dys	6 (1–15)
Stoma	Yes	4 (23.5)
	- colostomy	2 (50)
	- ileostomy	1 (25)
	- urostomy	1 (25)
	No	13 (76.5)
Intestinal anastomosis	Yes	11 (64.7)
	No	6 (35.3)
Enterocutaneous fistula	Yes	1 (5.8)
	No	16 (94.2)
Fascia closure	Yes	9 (53)
	No	8 (47)
Prothetic position	Onlay	2 (11.8)
	Inlay	1 (5.8)
	Retromuscolar	9 (52.8)
	Preperitoneal	0 (0)
	Intraperitoneal	5 (29.6)
Hospital stay	ICU	
	Mean (SD), dys	23.9 (±18.2)
	Median (range), dys	20 (2–71)
	Ward	
	Mean (SD), dys	28.4 (±21.2)
	Median (range), dys	23 (0–70)
Mortality rates	Peri-operative	5 (29.4)
	At 3 months	0 (0)
	At 6 months	0 (0)
	At 9 months	0 (0)

(*NPWT* negative pressure wound therapy, *pts* patients, *dys* days, *yrs* years, *SD* standard deviation, *ISS* Injury Severity Score, *MPI* Mannheim Peritoneal Index, *OA* Open Abdomen)

^a^More than one patient has more than one comorbidity

### Demographics characteristics

In Group 1, nine patients were enrolled: four of them were female. The mean age was 68.3 years (range 53–83). In all patients the surgical indication was incisional hernia repair. The use of the biological mesh was imposed due to the co-morbidities of the patients (grade 2 VHWG classification) and due to the presence of a previous wound infection and/or gastrointestinal tract resection (grade 3 VHWG classification). In six patients one or more comorbidities were present and listed as follows: immunodepression, a concomitant malignancy, abdominal aortic aneurism, diabetes, cardiopathy, bronchopneumopathy chronic obstructive. The others 3 patients have no comorbidity. However all these nine patients presented an intra-operative condition requiring the placement of a biological prosthesis: six patients underwent to colic anastomosis for colic resection, one patient had a prosthetic infection, and two patients had intra-abdominal infections (abscess from acute appendicitis and a ischemic small bowel).

In Group 2, four patients were enclosed (2 were female), with a mean age of 76.5 years (range 66–89). In this group the surgical indications were the following: abdominal hernia complicated with an enterocutaneous fistula, a diverticular perforation with a large incisional hernia, an eventration complicating a previous surgery with a small bowel resection and anastomoses, and an incisional hernia with a recto-vaginal fistula in Crohn disease. Two patients presented more than one co-morbidity: corticosteroid therapy, cardio-vascular disease, inflammatory bowel disease, and cerebrovascular disease. The other two patients had no previous diseases.

In Group 3, 17 patients were analyzed (9 patients were female). The mean age was 62.6 years (range 18–79). The main frequent comorbidities were: presence of cardiopathy and vasculopathy, the malignancy (gastric and colic cancer), and an immunosuppressed status. The others comorbidities present were the following: abdominal aortic aneurism, hemodialysis, and diabetes. Only three patients had no comorbidity. Seven patients (41.2 %) underwent to an OA procedure for intra-abdominal sepsis (anastomotic leak, appendicular peritonitis, biliary peritonitis), four patients (23.6 %) for intestinal ischemia, three patients (17.6 %) for trauma, two patients (11.8 %) after vascular surgery complications (duodenal perforation after exclusion of aortic aneurism), one patient (5.8 %) for severe acute pancreatitis. In 12 patients (70.6 %) the choice for the OA was to prevent the abdominal compartment syndrome; in 5 cases (29.4 %) the OA was performed to treat an increase of intra-abdominal pressure. In all patients the abdominal closure was planned at the first laparotomy and the decision of the biological prostheses usage was related to the intra-operative condition at the moment of the abdominal wall reconstruction. Eight patients (47 %) were from the Intensive Care Unit (ICU). For the three trauma patients the mean and median ISS were 38.3 (SD ± 14.3) and 35 (range 26–54) respectively. For the patient with pancreatitis the Balthazar score before surgery was E.

### Intraoperative details

In Group 1, the mesh was placed intra-peritoneally in three patients and in the retromuscolar plane in six patients.

In Group 2, the mesh was placed inlay (as a bridge between the medial edges) and onlay (in the subcutaneous space) in one patient each, and in the retromuscolar plane in two patients.

In Group 3, in 14 patients (the three trauma patients were excluded) the mean MPI was 26.6 (range 15–43). Four patients had a MPI < 21, five patients had a MPI between 21 to 29 and five patients had a MPI up to 29. According to the Björck score the majority of the patients (7 patients) had a grade Ia at the first laparotomy. Four patients had a grade Ib, and six patients had a grade IIb. At the closure, the rate of grade Björck Ia was in 13 patients (3 patients had a Björck IIa, and 1 patient a Björck IV). In 16 patients the OA was managed with a negative pressure wound therapy (NPWT) technique, and in 1 case the skin closure technique was performed. At the definitive intervention, the fascial closure was performed in nine patients (53 %), in the others eight patients only the posterior fascia was closed. In all 17 patients the abdominal wall was reinforced with a biological mesh: in nine patients the mesh was placed in the retromuscolar plane (sublay), in five patients intraperitoneally, in two patients onlay, and in 1 pt it was placed inlay. The time to definitive abdominal closure after OA was 6.1 days, with a range of 1–15. In four patients (25 %) a stoma was performed, meanwhile 11 patients (70.6 %) had an intestinal resection and anastomoses during the abdomen revision.

### Outcome

In two patients the mesh was removed: in the first case (Group 1) because the patient was re-operated for an intestinal ischemia, in the other patient (Group 3) for a duodenal fistula. In the last case the patient was left open and the wound was treated with a Vacuum Assisted Therapy (VAC).

In Group 1 the mean hospital stay was 21 days (range 7–70). The follow up at 3-months was completed for 9 patients: 2 patients has a grade I complication (wound infection), 1 patient had a grade IVb (re-operated for a small bowel ischemia), 1 patient died for a septic shock in a leucopenia, 4 patients were free from complications. One patient was lost at the follow-up. Four patients completed the follow-up at 6 months: 3 patients were free from complications, one patient died for a septic shock in leucopenia for the important lymphoproliferative disorder associated with recent chemotherapy regimen. One patient was lost at the follow-up. No patients completed the 9-months follow-up. One patient (11.1 %) died (septic shock in leucopenia).

In Group 2 the mean hospital stay was 21.5 days (range 7–45). One patient presented a grade I complication (wound infections), one patient presented a grade II complication (wound dehiscence) requires a vacuum-assisted closure technique. Two patients had no complications. One patient was lost to follow-up. The overall mortality was 0 %.

In the Group 3 the mean and median ICU stay was 23.9 days (range 2–71), and the mean ward stay was 28.4 days (range 0–70). In peri-operative time: 4 patients presented a grade I complication (wound infection), 1 patient presented a IIIb complications (a duodenal fistula due to a dehiscence of the duodenal perforation that required another surgical intervention and a VAC therapy to manage the fistula) and the patient is still hospitalized. Seven patients were free from complications. Five patients died (2 for aortic rupture, 2 for septic shock, and 1 for respiratory insufficiency and septic shock). Twelve patients completed the follow-up at 3 months: 1 patient presented a Grade II complication (incisional hernia non complicated), 1 patient require the pursuit of the VAC therapy to manage the duodenal fistula. Seven patients are free of complications. Three patients were lost to follow-up. No patients died at 3 months follow-up. Six patients completed the follow-up at 6 months: 1 patient presented a Grade II complication (incisional hernia non complicated). Two patients were healthy. Three patients were lost to follow-up. No patients died at 6 months follow-up. Two patients completed the follow-up at 9 months: 1 patient had no complications. One patient was lost to follow-up. No patients died at 9 months follow-up. The overall peri-operative mortality was 29.4 % (5 patients).

## Discussion

Incisional hernia remains a common complication in abdominal surgery and occurs after elective laparotomy with an incidence from 2 to 20 % or more [7, 19, 20]. However, these rates can increase (as high as 50 %) after abdominal closure in emergency surgery and in the abdominal repair following OA procedure [21–23]. The approach to the abdominal wall surgical repair is dramatically changed in the last years due to the availability of new repair materials, like biologic prostheses. Although in last years many studies tried to find an agreement about specific technique and materials that should be applied to repair of complex ventral hernia, at present, no strong consensus has been found yet [4, 24]. However the use of biologic prostheses is actually accepted by the medical community for addressing abdominal wall defects, particularly in contaminated and potentially contaminated fields [11, 25]. Despite this, the numerous advantages of biologic materials have been proven [11, 26, 27].

The present study reports a one-year experience about the usage of biological prostheses in different fields from elective surgery to emergency surgery and OA procedure. We analyze our surgical indications to the usage of biological meshes to evaluate the outcomes and the feasibility in these fields.

In the first group we considered only the patients underwent to ventral hernia repair in elective surgery. In all patients the abdominal wall defect was associated with high risk of contamination or an already existing infection imposed the positioning of a biological prostheses. The decision of the positioning of the prosthesis was based on the VHWG and the Italian Biological Prostheses work-group recommendations [4, 11], according to the degree of contamination potential or effective, and according to co-morbidities of the patient. In literature also others studies confirmed and supported these recommendations [7, 28, 29]. In our study, more than half of the patients of this group had more than one comorbidity (in particular immunosuppression and malignancy), that associated with a local wound infection or intra-abdominal infection have prevented the use of a synthetic mesh. In two third of patients the mesh was placed in the retromuscolar space, in line with the literature [9, 30]. The “sublay” position (posterior to the rectus muscle) has been the most used, in fact it allows for better tissue incorporation and lower chance of bacterial contamination, increases neo-angiogenesis and avoids the direct contact with intra-abdominal viscera [1, 9]. In this group only one patient died at three months for a septic shock, and in one case we had to remove the mesh because the patient was re-operated for an intestinal ischemia.

At the analysis of the second group (patients underwent to ventral hernia repair in emergency conditions) we found that the presence of wound infection or intra-abdominal infection are the main indication requiring a biological mesh positioning. This decision was also dictated by the important comorbidities present in half of patients. The other important point has been the age of patients (older than patients in others group, with a median age of 75.5 years). In last year the number of elderly patients (>65 years) underwent to ventral hernia repair in emergency setting is increasing [2]. A recent study reports that the risk of operative factors associated with the risk of surgical site infection were open procedures, incisional/ventral repairs, and hernia repairs with bowel obstruction/necrosis [2]. Into account of these data and according to the VHWG recommendations [4] we decided to repair the abdominal wall defect with a biological mesh, with favorable outcomes and with an acceptable hospital stay (median 16.5 days).

The third group was the largest group. The problem of abdomen closure after OA procedure remains the most critical point for these patients [21–23]. Data about long-terms outcome after repair of abdominal wall in OA procedure are still lacking and the durability of the different biologic meshes has not been clinically proved [26, 31]. Our surgical indications for an OA procedure requiring a biological prostheses positioning were the difficulty to close the abdominal wall (the anterior and the posterior fascia) or to reinforce the closure of the posterior fascia (when the anterior fascia was impossible to close). The main causes for a laparostomy were secondary peritonitis, intestinal ischemia and trauma. The conditions that required leaving the abdomen open remains the abdominal compartment syndrome (ACS) or the conditions that promote an intra-abdominal hypertension (IAH) (intestinal ischemia, acute severe pancreatitis, vascular complications), or in case of damage control surgery (trauma) [13, 26, 32–34]. Recently the approach to the intra-abdominal sepsis was changed and leaving the abdomen open in that condition has been proven to reduce mortality and post-operative complications [31, 35]. The primary goal remains the source control and a temporary abdominal closure was considered a valid instrument to manage severe abdominal sepsis [35]. In our series the traumatized patients undergone to OA had severe trauma (ISS > 25). In the two-third of cases we have proceed to OA management to prevent an ACS; in only one-third OA procedure was considered as treatment of the IAH or of the ACS. In patients with intra-abdominal sepsis the scoring system to estimate the severity and prognosis of secondary peritonitis we used was the MPI [15]. Literature reports that a MPI greater that 29 relates with high mortality rates (100 % vs 0–2.3 % in patients with MPI < 21) [15]. In our series one–third of patients have severe peritonitis (MPI > of 29), and these MPI values associated with the important comorbidities may be the responsible for the high peri-operative mortality rate (29.4 % at our analysis). In the present series the status of the OA has been evaluated at the first laparotomy and at the last one according to the Björck classification [18]. Data reported an increased number of patients presenting a Grade 1A (clean OA without adherence between bowel and abdominal wall or fixity) at the abdomen closure (from 41.4 %, at the first laparotomy, to 76.6 %, at the last laparotomy). These results showed the efficacy of the OA treatment in reducing the intra-abdominal contamination. Regarding to the technique used for temporary abdominal closure we used the NPWT in almost all cases. This strategy seems to be the best in preventing evisceration, actively remove any infected or toxic fluid from the peritoneal cavity, prevent the formation of entero-atmospheric fistulas, preserve the fascia and the abdominal wall domain, make reoperation easy and safe, and achieve early definitive closure [23, 26, 36]. Our goals were to close the abdomen wall as soon as possible (within 5–7 days) [26, 27, 37] to reduce the OA complications. A recent prospective controlled trial and a systematic review suggested that use of a dynamic fascial sutures combined with NPWT in the treatment of OA for intra-abdominal sepsis is safe and associated to a low rate of incisional hernias and entero-atmospheric fistula [22, 23]. However in our series almost all patients despite the NPWT alone, the closure of the abdominal wall happened in an average of 6.1 days, but in 53 % of cases the fascia was closed with a mesh in the retromuscolar space [9, 30]. The main complications after OA remain the enterocutenaous fistula and the long-term ventral hernia, in addition to the high rate of morbidity and mortality associated with the procedure [8, 26, 38]. A recent meta-analysis about OA and temporary abdominal closure techniques in non-trauma patients (4358 patients) reported a mortality rate near to 30 %, with an overall rate of fistula of 12.1 %, with a 14.6 % in series applying NPWT without fascial traction, and of 5.7 % after NPTW with fascial traction [38]. Others studies reported a high delayed facial closure rate ranging from 34 % and 73.6 % in septic patients, with lower rates in trauma patients due to the more frequent early abdominal closure [23]. In our study only one patient had a duodenal fistula and required the removal of the prostheses (being managed with a VAC therapy to control the spillage of duodenal contents [38]) and only one patient presented an incisional hernia without complications. These low rates of incisional hernia are probably due to the lack of a long-term follow-up. The other complications in present study were related wound infection or dehiscence that were managed with local wound dressing. Also in our data the rate of peri-operative mortality remains high (29.4 %), similar with the data find in the literature (30 %), however this is probably due to the extremely compromised conditions of the patients and the paucity of cases [2, 38].

Despite the limitations present study showed that the use of non-cross-linked porcine dermal meshes in different surgical setting, particularly in elderly and septic patients is safe and effective, without worsening morbidity and mortality.

## Conclusions

The present one-year prospective analysis shows that the use of swine dermal non cross-linked collagen prostheses for abdominal wall repair is safe and effective in general and emergency surgery, particularly in elderly and in immune-compromised patients. However more data are necessary. For this reason the development of big prospective studies are mandatory.
